# Frozen Blastocyst Embryo Transfer: Comparison of Protocols and Factors Influencing Outcome

**DOI:** 10.3390/jcm11030737

**Published:** 2022-01-29

**Authors:** Aikaterini Eleftheriadou, Abraham Francis, Mark Wilcox, Kanna Jayaprakasan

**Affiliations:** 1 School of Medicine, University of Nottingham, Nottingham NG7 2RD, UK; eleftheriadoukaterina1@gmail.com; 2 CARE Fertility, Nottingham NG8 6PZ, UK; francistharakan@yahoo.co.in (A.F.); Mark.Wilcox@carefertility.com (M.W.)

**Keywords:** frozen embryo transfer (FET), endometrial preparation, natural cycle, artificial cycle, pituitary downregulation, endometrial thickness

## Abstract

Background: Various factors, including treatment protocols, can influence the outcomes of frozen embryo transfers (FETs). The study objectives were to compare different endometrial preparation protocols of FET cycles and to evaluate the factors, including the endometrial thickness (ET), that affect outcomes. Methods: This observational cohort study involved 5037 women undergoing FETs at eight tertiary clinics in the UK between January 2016 and March 2019. The endometrial preparation protocols used were natural cycle (NC-FETs), artificial hormone support cycle with oestradiol valerate but without pituitary downregulation (AC-FETs) and artificial hormone support cycle with agonist downregulation (ACDR-FETs). Results: The mean (±SD) ages across NC-FET, AC-FET and ACDR-FET groups were 36.5 (±4.2), 35.9 (±5.0) and 36.4(±4.9) years, respectively. LBRs were comparable (40.7%, 175/430; 36.8%, 986/2658; and 36.7%, 716/1949, respectively) across the three groups. Clinical pregnancy, implantation, multiple pregnancies, miscarriage and ectopic pregnancy rates were also similar. In the regression analysis of variables including age, duration of infertility, number of embryos transferred, protocol type and endometrial thickness, age was the only significant predictor of LBRs, although its predictive ability was poor (AUC: 0.55). With the overall LBR of the study population being 37.1%, the post-test probability of a live birth at an ET of <5 mm was 0%, and at 5–5.9, 6–6.9, 7–7.9 and 8–8.9 mm, the probabilities were 16.7%, 33.8%, 36.7% and 37.7%, respectively. The LBR remained above 35% up to the 14–14.9 mm range and then declined gradually to 23% for the 17–25 mm range. Conclusions: The FET outcomes were similar for the three protocols used for endometrial preparation. The protocol type and endometrial thickness were not predictive of FET outcomes; age was the only predictive variable, despite its low predictive ability.

## 1. Introduction

Over a third of the IVF cycles in the UK are Frozen-thawed Embryo Transfers (FETs). Success rates of fresh and frozen embryo transfer cycles are now comparable [1]. Elective freezing of embryos and potentially subsequent FETs are now commonly employed for hyper-responders with increased risks of developing ovarian hyperstimulation syndrome (OHSS) [2,3], preimplantation genetic testing (PGT) [4], fertility preservation for health or social reasons and uterine pathologies detected in the course of treatment [5]. Further, some units across the world have adopted a gradual shift from fresh transfers to elective freezing and subsequent FETs for the convenience of batching IVF cycles and due to the possibility of controlled ovarian stimulation compromising implantation and pregnancy outcomes [6], coupled with improved freezing techniques leading to enhanced success rates with FETs [7].

The success of FET cycles is dependent on the synchronisation of the endometrium to be receptive for the embryo [8]. In order to prepare the endometrium, several protocols have been suggested: natural cycles, where detecting the luteinising hormone (LH) surge, and, therefore, the ovulation, defines the timing of the transfer; modified natural cycles, where ovulation is triggered by the administration of human chorionic gonadotrophin (hCG); artificial cycles, with the support of exogenous hormones with or without the addition of gonadotrophin-releasing hormone (GnRH) agents to temporarily suppress ovarian function and, finally, cycles with ovulation induction with drugs [9]. The efficacy and the safety of these FET protocols have been examined by multiple studies, but there is a lack of consensus on how the endometrium should be prepared and synchronised [9,10,11,12,13,14,15,16,17,18,19].

FET protocols prime the endometrium for the implanting of an embryo, and endometrial thickness is evaluated to assess how well the endometrium is prepared [20,21,22]. In fact, endometrial thickness in FET treatments determines the timing of the administration of luteal support and of the transfer [23]. The incidence of thin endometrium is linked with poor prognoses for live births in FET treatment cycles, and most studies set the optimum cut-off value of thickness at 7 or 8 mm [24,25,26], although there is still an ongoing debate on how thick is thin. Hence, the efficiency of endometrial thickness as a prognostic factor for pregnancy outcomes, assisting clinicians in evaluating the possibilities of conception, has been investigated. These investigations have shown poor predictive accuracy but a strong association between improved results and thicker endometrium [27,28,29,30,31].

The main objective of this study was to compare different protocols of endometrial preparation for FET cycles with live birth rates and maximum endometrial thickness as the main outcome measures. We have also evaluated live birth rates following FETs at different cut-off levels of endometrial thickness to define the optimal cut-off value, assessing its predictive accuracy.

## 2. Methods

### 2.1. Patient Population and Recruitment

This observational cohort study involved 5037 women undergoing conventional blastocyst FET cycles at eight tertiary fertility clinics in the UK between January 2016 and March 2019. Only one cycle (the first FET cycle) per participant was included. All subjects had BMIs of ≤35 Kg/m^2^. FETs of pre-implantation genetic testing (PGT) cycles were excluded. The data were obtained from the prospectively recorded computerised database. Study approval was obtained from the institutional review board. For the purpose of the study, the data were anonymised throughout.

### 2.2. Embryological Data

The stage of the embryos for the FETs was the blastocyst stage. The number of embryos transferred was one or two (mostly single embryo transfers).

### 2.3. Endometrial Preparation

The endometrial preparation protocols used were natural cycle (NC-FET), artificial hormone support cycle with oestradiol valerate but without pituitary downregulation (AC-FET) and artificial hormone support cycle with agonist downregulation (ACDR-FET).

In natural cycles (NC-FET), monitoring with ultrasound examinations and ±LH blood tests was commenced on day 7 or 8 of the cycle, after spontaneous menses, to monitor follicular development, conduct endometrial assessments and find out the timing of the LH surge. Subsequent monitoring visits were scheduled depending on the initial assessment. When the LH surge and ovulation were detected, the blastocyst embryo transfer was scheduled. Progesterone vaginal pessaries commenced on the day of the embryo transfer.

In artificial hormone support cycles (AC-FET), oral oestrogen (oestradiol valerate, 2 mg three times a day) was commenced on the first day of menstruation. After 10–12 days, an ultrasound examination was undertaken to assess endometrial thickness. When the thickness reached ≥7 mm, the supplementation of vaginal progesterone (Utrogestan, 400 mg twice daily) started on day 15 ± 2, and the embryo transfer was planned on day 20 ± 2, while oestradiol was continued at the same dose. If the endometrial thickness was <7 mm, the oestradiol dose was increased to 8–12 mg per day, and after 5–7 days, the ultrasound evaluation was repeated. If the criteria were still not met, depending on the past treatment history, cycle cancellation or treatment continuation was discussed, and a joint decision whether to pursue FET or cycle cancellation was made. 

The third type of protocol (ACDR-FET) incorporated pituitary downregulation with commencing GnRH agonists (Busereline, 0.5 mg per day) from the mid-luteal phase of the cycle immediately prior to the planned FET cycle. Two weeks after commencing GnRH agonists, a transvaginal scan ± oestradiol blood test was performed to confirm downregulation. Once downregulation was confirmed, oestradiol was commenced as in the AC-FET protocol. Subsequent protocols are similar to the AC-FET protocol as described above, and daily GnRH agonists were continued until the day of progesterone commencement.

In all women, embryo transfers were carried out by experienced clinicians using the soft Wallace^®^ Sure View^®^ (CooperSurgical Fertility Solutions, Knardrupvej, Denmark) Catheter under ultrasound guidance. Urine pregnancy tests were conducted 13–14 days following the embryo transfer. Early pregnancy scans were arranged 4 weeks from positive pregnancy tests. Luteal support with progesterone in natural cycle cases and both Oestrogen and progesterone in artificial cycles were continued for 10 weeks of gestation if pregnant.

### 2.4. Primary and Secondary Outcomes

The primary outcome was live birth rates per transfer, described as the delivery of at least one live baby at or after 24 weeks gestation. Secondary outcomes included biochemical pregnancy, clinical pregnancy, implantation, ectopic pregnancy and miscarriage rates. Biochemical pregnancy was defined as a positive urine pregnancy test, while clinical pregnancy was defined as a viable intrauterine pregnancy on ultrasound scan. Miscarriage was diagnosed for all pregnancy losses after a confirmed biochemical pregnancy. Only the data from those who had embryo transfers were analysed, excluding cancelled FET cycles.

## 3. Statistical Analysis

Statistical Package for Social Sciences software (SPSS Version 26, IBM Corp., Armonk, NY, USA) was used for statistical analysis. The distribution of the data was checked for normality by the application of the Kolmogorov–Smirnov test. The demographic data and the outcome data of the different groups were compared using the Student’s *t*-test or Mann–Whitney *U* test for continuous variables depending on the statistical distribution of the data. The chi-square test was used for comparing the dichotomous variables. *p*-values < 0.05 were considered statistically significant. Logistic regression analysis was performed to assess the effect of each independent variable, including the type of protocol, on the chances of live births. Receiver–operating characteristics (ROC) curve analysis was performed to quantify the ability of endometrial thickness to discriminate between subjects who had successful (live births) and those with unsuccessful FET outcomes. The sensitivity, specificity, positive and negative predictive value and post-test probabilities for live birth at different cut-off values of endometrial thickness were calculated.

## 4. Results

Overall, the analysis included 5037 FET cycles, performed with three different protocols. A set of 430 cycles were carried out with the natural cycle regime (NC-FET), 2658 artificial hormone support cycles with oestradiol valerate (AC-FET) but without downregulation were carried out and 1949 artificial hormone support cycles with agonist downregulation were carried out (ACDR-FET). The mean ages (±SD) of patients across NC-FETs, AC-FETs and ACDR-FETs were 36.5 (±4.2), 35.9 (±5.0) and 36.4 (±4.9) years, respectively (*p* < 0.01). The mean durations of subfertility (±SD) across these groups were 1.63 (±1.4), 2.56 (±2.3) and 2.32 (±2.2) years, respectively (*p* < 0.01). Interestingly, the number of embryos transferred was shown to be significantly different in the comparison of NC-FET (1.2 ± 0.4), AC-FET (1.13 ± 0.3) and ACDR-FET (1.16 ± 0.4) (*p* < 0.01).

The pregnancy outcome measures in our study population are shown in Table 1. The overall LBR in the study was 37.1% (1869/5037). LBRs were comparable (40.7%, 175/430; 36.8%, 976/2658 and 36.7%, 716/1949, respectively) across all three protocol groups. Subgroup analysis based on the number of embryos transferred led to comparable results, with no difference in LBRs across the FET protocols in single (41.2%, 141/342; 36.4%, 842/2316 and 36.1%, 587/1625, respectively; *p* = 0.19) or double embryo transfer (39.3%, 33/84; 41.1%, 138/336 and 40.1%, 126/314, respectively; *p* = 0.94). Further subgroup analysis was conducted to compare the live birth rates depending on the stage of the embryos transferred. A total of 86.6% (5035/5813) were blastocyst transfers, and 13.4% (778/5813) were cleavage-stage embryo transfers. The live birth rates, which were 40.9% (175/430 NC-FETs), 36.9% (978/2654 AC-FETs) and 36.7% (716/1951 ACDR-FETs), were similar (*p* = 0.23) for blastocyst-stage transfers. The live birth rates were low for cleavage-stage FETs overall, with 1.9% (2/107) for NC-FETs, 4.7% (18/387) for AC-FETs and 10.6% (30/284) for ACDR-FETs (*p* < 0.01). 

In the multiple logistic regression analysis incorporating age, duration of subfertility, type of protocol, maximal endometrial thickness and number of embryos transferred, the type of protocol was not a significant predictor of live births. Age (OR 0.969, 95% CI: 0.956–0.981) was the only significant predictor of LBRs. However, the discriminative ability of age to predict live births was poor, as indicated by the area under the curve (AUC) of 0.55 (95% CI: 0.54–0.57) on the ROC curve analysis. Endometrial thickness was not predictive of live birth rates. 

**Figure 1:**
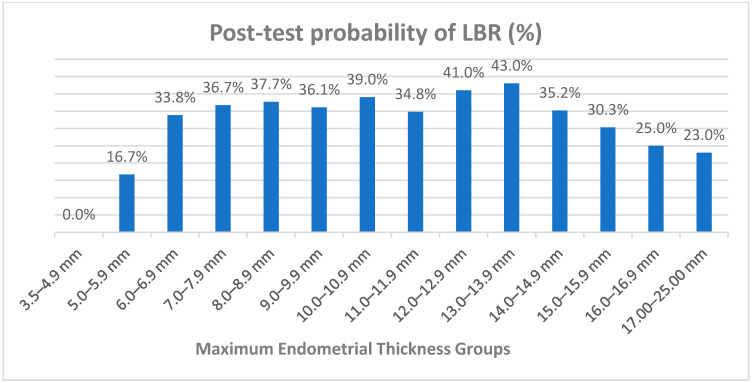
Post-test probability for live birth rates (LBR) for different cut-off values of the maximal endometrial thickness.

The sensitivity, specificity, positive and negative likelihood ratio and the post-test probability of live birth at different endometrial thicknesses are shown in Table 2. With the overall LBR of the study population being 37.1%, the post-test probability of a live birth at an ET of <5 mm was 0%, and at 5–5.9, 6–6.9, 7–7.9 and 8–8.9 mm, the probabilities were 16.7%, 33.8%, 36.7% and 37.7%, respectively. LBRs remained above 35% up to the 14–14.9 mm range and then declined gradually to 23% for the 17–25 mm range. The highest post-test probability (43%) was at an endometrial thickness of 13.0–13.9 mm (Figure 1). 

## 5. Discussion

In the present study, the data indicate that live birth rates following blastocyst FET treatment were similar for all of the three protocols (natural cycle and artificial hormone support cycle with and without GnRH agonist downregulation) used for endometrial preparation. In the subgroup analysis, live birth rates were comparable across the three treatment groups regardless of women’s ages and the number of embryos transferred. Implantation rates, biochemical pregnancy rates, clinical pregnancy rates and multiple pregnancy rates were also similar across all of the three groups. To our knowledge, this is the largest study reported in the literature to include the comparison of three different FET protocols in 5037 frozen cycles from a fertility tertiary centre.

In concordance with our study, the most recent Cochrane dataset review regarding the regimes for FET endometrial preparation, based on 31 RCTs and including 5426 women, concluded that there was not adequate evidence to suggest any specific protocol for FET endometrial preparation. While agonist downregulation was suggested to probably improve live birth rates compared to artificial cycle FETs without downregulation, clinical pregnancy rates were similar across the groups [12]. While other studies, including systematic reviews, meta-analyses and RCTs, present contradictory results, there have been reports of a lack of high-quality data [32,33,34,35,36,37,38,39,40,41]. Although a prospective RCT is recommended and ideal, we believe that our large study, with its analysis of prospectively collected data, provides robust evidence on the subject.

In a prospective study of 570 FET cycles, comparing true and modified NC-FETs with AC-FETs, similar live birth rates were found, but higher miscarriage rates were the result of artificial cycles [34]. In our study, we used the same dose of oestradiol valerate (6 mg) from day 1 that may have influenced better suppression of follicular development and excessive LH secretion [42], while Cerillo et al. applied an incremental dosage. Tomas et al. reported higher biochemical pregnancy rates and pregnancy loss rates in AC-FETs but similar clinical pregnancy and delivery rates when compared with NC-FETs with luteal support. Luteal support was given for a period of 14 days only regardless of the result of the pregnancy test [43]. Higher biochemical rates in hormone replacement cycles but similar implantation rates, as in the present study, were also seen in another retrospective study in IVI group clinics with 4525 cycles, evaluating NC-FETs and AC-FETs with GnRH downregulation [44]. Van de Vijver et al. examined the efficacy of adding GnRH agonist downregulation in 1129 artificial cycles and found comparable live birth rates [45]. On the other hand, in a study in 2016 with endometrial transcriptome analysis, NC-FETs were shown to be superior to AC-FETs in patients with recurrent implantation failure [46]. Melnick et al., utilising trophectoderm biopsies and 24-chromosome screening, evaluated that oestradiol peak levels were lower in natural cycles, suggesting that natural regimes in ovulatory women resulted in higher live rates than hormone support cycles in anovulatory women [47]. Almost all the published studies have reported data from the transfer of embryos at the cleavage stage as well as the blastocyst stage. However, we have included data from only blastocyst FETs, which has limited the bias relating to the influence of embryo quality on treatment outcomes. We feel that women having blastocyst FETs would be the ideal population to test the effects of various endometrial preparation protocols on FET outcomes; therefore, the evidence from this study is likely to be robust.

NC-FET is considered patient-friendly in women with regular cyclicity, including no medical intervention, but it needs monitoring to detect LH surges and ovulation. Urinary LH kits increase the convenience of the protocol by reducing extensive monitoring and frequent visits to clinics [19,48,49]; however, it has limitations due to the variability of LH surges in configuration, amplitude and duration [50]. Therefore, fewer clinicians and patients prefer NC-FET protocols, which is reflected in our study with the smaller sample size in the NC-FET group compared to the other two study groups. AC-FET treatment cycles are often preferred, as they can be used for all women regardless of their menstrual cyclicity and can easily be scheduled, thus benefitting the planning of workloads in clinics and patients’ preferences [16,51]. However, this approach poses some disadvantages, such as its additional cost, patients’ discomfort and potential side-effects of oestrogen supplementation, such as thrombotic risks, nausea or increases in weight [14,45]. The addition of the GnRH analogue to guarantee pituitary suppression and avoid early exposure of endometrium to progesterone can induce hypoestrogenic side effects, fatigue, headaches and some anxiety in patients due to prolonged treatment and additional medication [39,52]. While the outcomes of different protocols of FET have been reported to be similar in many studies, including ours, the first RCT evaluating the cost-efficiency of modified NC-FETs and AC-FETs, expenses were found to be comparable [15].

In the setting of FET cycles, thin endometria have been linked negatively with pregnancy outcomes. In fact, our data suggested that live birth rates are predicted to be low at ≤6 mm of endometrial thickness and 0% in the group of <5 mm. However, it is important to note that about 17% and 33.8% of women with endometrial thicknesses of 5–5.9 mm and 6–6.9 mm, respectively, had a live birth. This information is useful for counselling women before cancelling treatment cycles altogether in those women. A number of studies propose results similar to ours, associating thin endometrium with poor live birth rates [24,25,53]. Liu et al. suggested that pregnancy outcomes decreased for every 1 mm decrement below 7 mm in FET cycles. Interestingly, the threshold was lower compared to fresh embryo transfers, in which the decrease started at 8 mm, probably due to endometrial advancement or changes in receptivity caused by ovarian stimulation, leading to different hormonal milieu [26]. Thin endometrium may result from any endometrial destruction that leads to adhesions and scarring or be idiopathic [54]. Poor angiogenesis and reduced blood flow have also been suggested to cause decreases in thickness [55,56]. The non-responsive thin endometrium is considered to bring the embryo closer to basal layer spiral arteries and high-oxygen tension areas, leading to lower implantation rates [57], but also to other pregnancy complications [58].

Our data also indicated low pregnancy rates in extremely high endometrial thickness as well, especially after the group of ≥16 mm, from which point they showed to decrease. Bu et al. supported that clinical pregnancy and live birth rates were greater in the group of above 14 mm of endometrial thickness in patients over 35 years old. However, the number of those was approximately 2% of the total [24]. Check et al. similarly advocated that thicker endometrium > 14.5 mm does not compromise FET success [23]. Both studies defined the thickness threshold at 14 mm, at which our data also indicate a probability of live birth of 31.53%; however, above this value, rates significantly decreased. It has been suggested that abnormally thickened endometria affect pregnancy outcomes due to trauma from the transfer catheter or unsupportive histologic patterns [59]. Studies have also evaluated other conditions that this could be involved in, such as hyperplasia and intrauterine pathologies, including polyps and fibroids [60,61,62].

In a review and meta-analysis with IVF cycles, endometrial thickness was shown to have a limited capacity to predict pregnancy [30]. In this study, the data presented a strong association of gradually thicker endometrium with better pregnancy rates for every cut-off. Some other studies reached similar conclusions, with endometrial thickness constituting one of the most valuable and investigated markers for endometrial receptivity [28]. While several studies propose low predictive accuracy for pregnancy outcomes [28,63], conflicting reports on the effects of extreme endometrial thickness on treatment outcomes may possibly be related to a number of confounders, including female age, the number of embryos transferred or retrieved oocytes that affect outcomes [64,65].

## 6. Strengths and Limitations

The strength of this study is its large size, analysing a total of 5813 FET cycles and being multi-centred. We have also made an effort to reduce the effects of numerous confounding factors by including only the first FET cycle performed. The limitation of this study is that it is of retrospective and non-randomised design, and this may have caused some bias. However, the data are prospectively recorded; therefore, the data are robust and accurate.

## 7. Conclusions

The FET outcomes were similar for all of the three protocols used for endometrial preparation. The selection of the protocol for endometrial preparation for FETs can therefore be dictated by the convenience of the patients and the fertility unit. Endometrial thicknesses were not predictive of FET outcomes; age was the only predictive variable, albeit its low predictive ability. While extremely thin endometria seem to be associated with lower live birth rates, it is important to note that about 31% of women had a live birth with an endometrial thickness of 5–6.9 mm.

## Figures and Tables

**Table 1:** Pregnancy Outcomes and clinical characteristics of natural cycle (NC-FET), artificial hormone support cycle without downregulation (AC-FET) and artificial hormone support cycle with agonist downregulation (ACDR-FET) groups.

	NC-FET (1)	AC-FET (2)	ACDR-FET (3)	*p*-Value
Maximum Endometrial Thickness (mm) ^a^	9.5 ± 1.95	9.4 ± 1.7	9.9 ± 1.9	<0.001 ^d^
Live Birth ^b^	175/430 (40.7%)	978/2658 (36.8%)	716/1949 (36.7%)	0.27 ^c^
Clinical Pregnancy ^b^	206/430 (47.9%)	1121/2658 (42.2%)	865/1949 (44.4%)	0.052 ^c^
Implantation ^a^	0.46 ± 0.5	0.44 ± 0.49	0.46 ± 0.49	0.56 ^d^
Biochemical Pregnancy ^b^	246/430 (57.2%)	1476/2658 (55.5%)	1136/1949 (58.3%)	0.17 ^c^
Miscarriage ^b^	36/243 (14.8%)	252/1476 (17%)	223/1136 (19.6%)	0.1 ^c^
Multiple Pregnancy ^b^	15/206 (7.3%)	55/1121 (4.9%)	53/865 (6.1%)	0.28 ^c^
Ectopic Pregnancy ^b^	1/246 (0.4%)	6/1476 (0.4%)	4/1136 (0.35%)	0.97 ^c^

^a^: values are mean ± standard deviation; ^b^: values are percentages; ^c^: Chi-square test was used for dichotomous variables; ^d^: Kruskal–Wallis test was used for continuous variables. Mann–Whitney test was also used for continuous variables. Maximum Endometrial Thickness *p*-value 1–2 > 0.05; *p*-value 1–3 < 0.05; *p*-value 2–3 < 0.05. Implantation *p*-value 1–2 > 0.05; *p*-value 1–3 > 0.05; *p*-value 2–3 > 0.05. *p*-value < 0.05 is considered statistically significant.

**Table 2:** Accuracy measures for cut-off values of the maximal endometrial thickness (MET) based on their ability to predict live births as outcomes following FET treatment.

Cut-Off Values (mm) for Live Birth	No. of Cycles	Sensitivity (%)	Specificity (%)	LR+ (95% CI)	LR− (95% CI)	Post-Test Probabilities If Test Positive (%)
3.5–4.9	5	0	99.8	0.00	1.0	0.00
5.0–5.9	12	0.1	99.7	0.34	0.99	16.7
6.0–6.9	65	1.2	98.6	0.87	0.99	33.8
7.0–7.9	354	6.9	92.9	0.98	0.92	36.7
8.0–8.9	1213	24.4	76.1	1.02	0.68	37.7
9.0–9.9	969	18.7	80.4	0.96	0.77	36.1
10.0–10.9	681	14.2	86.9	1.09	0.84	39
11.0–11.9	394	7.3	91.8	0.90	0.92	34.8
12.0–12.9	236	5.2	95.6	1.18	0.95	41
13.0–13.9	123	2.8	97.8	1.28	0.97	43
14.0–14.9	54	1.0	98.9	0.92	0.99	35.2
15.0–15.9	33	0.5	99.3	0.73	0.99	30.3
16.0–16.9	12	0.2	99.7	0.56	1.00	25
17.0–25.0	13	0.2	99.8	0.51	1.00	23

LR+: Likelihood ratio of a positive result; LR−: likelihood ratio of a negative result. Pre-test probability was 37.1%.

## Data Availability

The authors confirm that the data supporting the findings are available from the corresponding author upon reasonable request.
